# Pulmonary stenosis development and reduction of pulmonary arterial hypertension in atrioventricular septal defect: a case report

**DOI:** 10.1186/1749-8090-4-49

**Published:** 2009-09-16

**Authors:** Emeline Barth, Hélène Bouvaist, Stéphanie Marlière, Gérard Ninet, Gérald Vanzetto

**Affiliations:** 1 Clinique de cardiologie, CHU de Grenoble, BP 217, 38043 Grenoble cedex 09, France

## Abstract

A 24-year-old patient was admitted for dyspnoea and syncope. He had a previous history of complete atrio-ventricular septal defect and trisomy 21. At the age of 6 months, in 1984, cardiac catheterization revealed a quasi-systemic pulmonary arterial hypertension with a bidirectional shunt corresponding to an Eisenmenger syndrome. Corrective cardiac surgery was not performed at this time because surgical risk was considered too high. Until the age of 20 years old, he showed few symptoms while under medical treatment. But since 2006, his functional status became worse with an increased dyspnoea, syncopes, and severe cyanosis. In these conditions, haemodynamic parameters have been re-evaluated in 2006 and 2008.

They highlighted a late and progressive development of a valvular and infundibular pulmonary stenosis leading to a normalisation of pulmonary arterial pressures. At the age of 24 , the patient underwent corrective cardiac surgery which was successful. Late development of both infundibular and valvular pulmonary stenosis have not been described before in non operated congenital ventricular septal defects, but development of one or the other abnormality would be found in 8% of patients. The physiopathological mechanism of this obstruction is unclear. Nevertheless, in unoperated congenital cardiac shunt lesions, reversibility of severe pulmonary arterial hypertension should be reconidered and re-assessed during follow up.

## Case presentation

A 24 year-old-man, was admitted to hospital for repeated syncopes and increased dyspnea. He was treated for a severe pulmonary arterial hypertension (PAH), secondary to atrio-ventricular septal defects (AVSD) associated with trisomy 21. Indeed, the first cardiac catheterization was performed in 1984 at the age of 6 months, at Robert Debré Hospital, and confirmed the "Eisenmenger syndrome" associated with a large posterior and complete atrio-ventricular septal defect, with grade 1 mitral regurgitation. Right-to-left shunt was moderate and the left-to-right still predominant. Left ventricular systolic pressure was 85 mmHg and pulmonary artery (PA) systolic pressure almost systemic at 75 mmHg with pulmonary to systemic vascular resistance ratio of 0,3, and pulmonary-systemic outflow ratio of 2,7. The right ventricular (RV) systolic pressure was 85 mmHg so catheterization showed at this time a non significant RV-PA outflow gradient of 10 mmHg. Right heart was very dilated and non hypertrophic. At physical examination, the young boy had a good psychomotor and weight development, no cyanosis and no sign of cardiac failure under digitalo-diuretic treatment. Pulmonary resistance was considered in the borderline operable range values but spontaneous prognosis was estimated equal to post-operative prognosis so corrective surgery was not proposed.

Treatment digitalo-diuretic was stopped in 1987. Few cardio-respiratory complications occurred during his childhood, and cyanosis was moderate with a good exertion capacity until 2004.

By then, his functional status started becoming worse with progressive increased dyspnea, pulmonary infections, cyanosis due to a severe chronic hypoxemia and secondary erythrocytosis.

**Figure 1:**
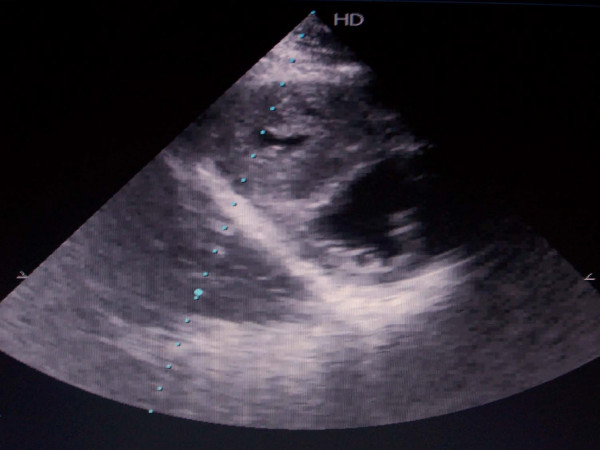
**Echocardiography -subcostal view: hypertrophic right ventricule**.

**Figure 2:**
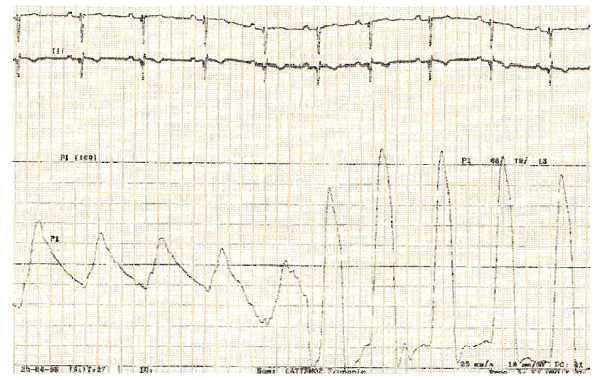
**Cardiac catheterization 2006: systolic pulmonary arterial pressure of 60 mmHg and right ventricular pressure of 105 mmHg so outflow gradient obstruction AP-RV of 45 mmHG**.

Taken in charge at Grenoble Hospital in 2006, oxygen saturation was 80%, NYHA functional class II-III, without any sign of cardiac failure. The 6 minutes walking test was 180 m. Echocardiography found a persistant complete atrio-ventricular defect of 2 cm with moderate mitral regurgitation, but revealed a non dilated hypertrophic RV (figure 1) associated with a mixed, infundibular and valvular, pulmonary stenosis (PS) with RV-PA outflow gradient of 60 mmHg. Cardiac catheterization was checked (figure 2) and showed a decreased but still relatively high pulmonary arterial pressure (systolic 60 mmHg -diastolic 25 mmHg- mean 40 mmHg) with a RV-PA obstruction at 45 mmHg. Shunt was bidirectional and cardiac output was normal. Under vasoreactivity test (nitric oxide drug), pressures slightly decrease to 54- 23-38 mmHg. Vasodilator treatment was started with prostacyclins and endothelin inhibitors (Sildenafil 20 mg × 3 per day and Bosentan 125 mg twice a day).

**Figure 3:**
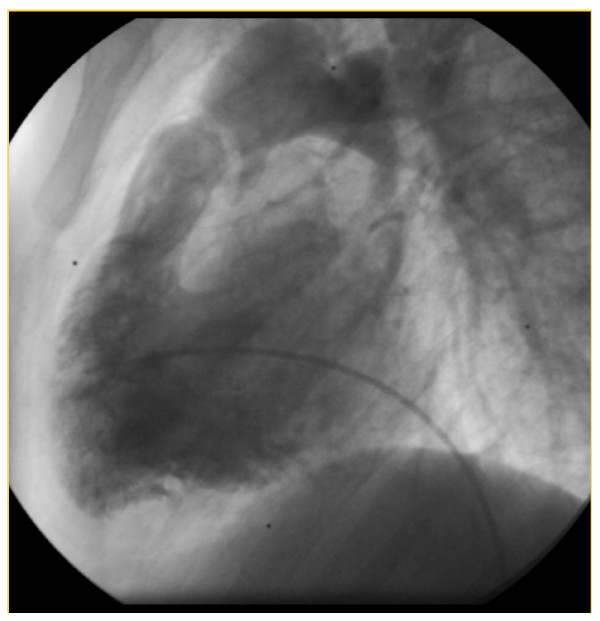
**Right ventricular angiography 2008: Description: normal anatomic pulmonary artery**.

**Figure 4:**
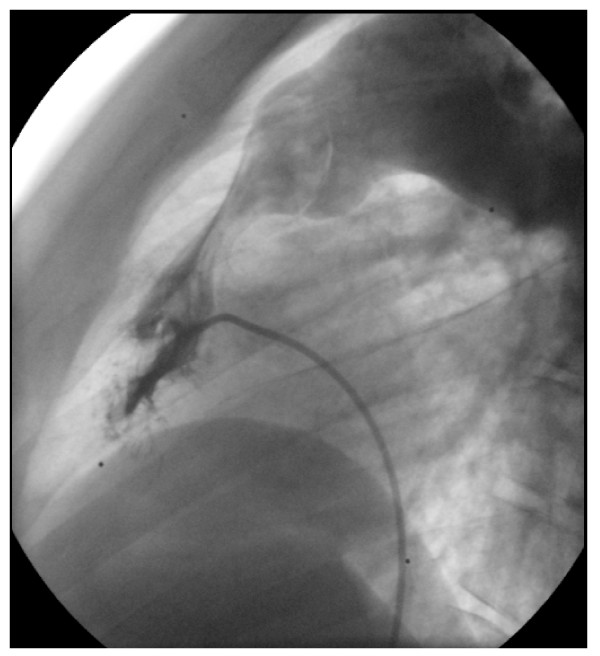
**Right ventricular angiography 2008: dynamic infundibular pulmonary artery stenosis during blood flow ejection**. Proximal and distal pulmonary artery diameter = 28 mm.

**Figure 5:**
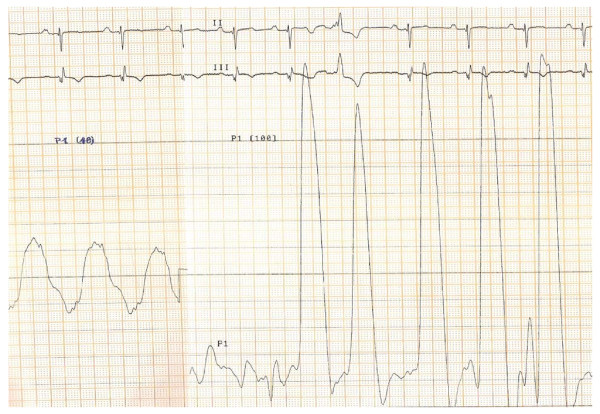
**Cardiac catheterization 2008: increased of pulmonary stenosis: systolic pulmonary arterial pressure of 27 mmHg and right ventricular systolic pressure of 126 mmHg so outflow gradient obstruction AP-RV of 99 mmHg**.

In 2008, he was readmitted in hospital because of repeated syncopes and major dyspnoea. Oxygen saturation was 75%, there was no fluid retention. Echocardiography revealed an increased RV-PA obstruction of 82 mmHg with severe RV hypertrophy. Catheterization parameters confirmed the severity of pulmonary obstruction with pullback pressure tracing from the PA to the RV measuring a 90 mmHg outflow gradient obstruction. The mechanism of stenosis was explained by angiographic imagery (figures 3 and 4) showing a dynamic infundibular pulmonary stenosis and a severe valvular stenosis. PAH further decreased to quasi-normal pulmonary pressure (systolic 37 mmHg- diastolic 13 mmHg- mean 20 mmHG) with a normal cardiac output and low pulmonary vascular resistance of 2 Wood units (figure 5).

The young man benefited from a successful corrective cardiac surgery in 2008 performed by Pr NINET (Louis Pradel Hospital in Lyon, France). Pulmonary valvar was strongly calcified and narrow. Outflow tract was muscular and thick. The surgeon closed the atrial and ventricular parts of the ASVD, replaced the pulmonary valve with a biological prothesis, and reconstructed 6 cm of pulmonary infundibulum. Patient needed a pacing implantation because of a paroxystic atrio-ventricular bloc in the post-operative period. He is today asymptomatic. Post-operative echocardiography found a RV-PA residual outflow gradient of 12 mmHg, a mean PA pressure of 26 mmHg and no residual shunting.

## Discussion

AVSD account for approximately 3% of all congenital heart diseases, frequently associated with other malformations such as in trisomy 21 [1]. Additional cardiac abnormalities are found in about 20% of congenital AVSD cases during follow up (S Glen and J Burns prevalence study [2]). Most of them are detected at the initial assessment but PS may develop subsequently. The most frequent malformation is infundibular PS which represents 5,8% of AVSD and is often detected in adults. Pulmonary valvular stenosis would represent about 2% of cases and is rather found in paediatric follow up.

The natural history of large AVSD is the evolution to the "Eisenmenger syndrome", which refers to the development of bidirectional or predominant right-to-left shunt accompanied by oxygen-unresponsive hypoxemia due to a severe PAH [3]. Surgery is supposed to be performed before pulmonary vascular changes are "fixed". Therapeutic option relies on catheterization-based calculations of pulmonary blood flow, pulmonary vascular resistance and responses to acute vasodilator testing to assess PAH reversibility and likelihood of surgical success. Unfortunately, haemodynamic parameters of unoperated patients are not hemodynamically re-assessed and surgery never reconsidered during follow up.

Some cases of "masked" infundibular pulmonary obstruction in young patients have been previously reported in ventricular septal defect with severe pulmonary hypertension by J Vogel and S Blount in 1965 [4]. Catheterization parameters of 32 patients with ventricular septal defect and PAH responded to vasodilator tests, with pullback pressure tracing from PA to RV available, have been reviewed. 27 of these patients manifested some degree of infundibular obstruction after administration of talazoline. Four of the 27 patients presented an associated atrial septal defect. One of them had a history similar to this case report. At the age of 1 year old, he presented a systemic right heart pressure with no spontaneously detected right sided gradient, but a "masked" 25 mmHg infundibular gradient under talazoline. Then, he developed upon catheterizations a 57 mmHg gradient pulmonary stenosis with a significant decrease in pulmonary arterial pressure from 87 to 57 mmHg. He finally underwent a corrective surgery at the age of 24 years old and one year later, his right sided pressure were perfectly normal. The surgeon did not find any outflow tract or valvular abnormality so they concluded a dynamic gradient. For the other cases, no solid arguments concerning the nature of obstruction, organic or functional, could be explained, even though functional mechanism remains the main hypothesis. This study suggested that "masked" infundibular obstruction may exist from birth. This is rarely obvious at the initial assessment because of the significant distal vascular bed obstruction and high RV pressure so means must be employed to render evident. First, try to decrease the distal obstruction and then obtain pullback pressure tracings from AP to RV. Further development of organic or dynamic PS may reduce pulmonary blood flow and protects lungs from vascular damages, so corrective surgery could be reconsidered, even in adults. This observation suggests by the way, that in children, surgical thresholds should be reviewed and tested in controlled studies. At initial shunt mediated PAH assessment, in newborn period or in childhood, some haemodynamics parameters are considered beyond surgical thresholds or borderline cases, and never re-assessed. So surgery is often never reconsidered. However children often have a more reactive pulmonary vascular bed than the adults and the prevalence of acute vasoreactivity is higher. So when are obstructive vascular changes really "fixed"?

Most centers tend to rely on catheterization-based calculations of pulmonary blood flow resistance and pulmonary pressure to assess PAH reversibility and likelihood of surgical success, with a better surgical outcome if pulmonary vascular resistance is below 15 Woods units and pulmonary-systemic resistance ratio below 2/3. This attitude remains the consensus of opinion based on surgical experience rather than rigorously tested standards.

## Abbreviations

PAH: pulmonary arterial hypertension; PA: pulmonary artery; RV: right ventricular; AVSD: atrio-ventricular septal defects; PS: pulmonary stenosis.

## Consent

Written informed consent was obtained from the patient for publication of this case report and accompanying images. A copy of the written consent is available for review by the Editor-in-Chief of this journal.

## Competing interests

The authors declare that they have no competing interests.

## Authors' contributions

EB wrote the manuscript. HB performed the two last cardiac catheterizations. GN performed the surgery. GV and SM supervised manuscript redaction.

All the authors read and approved the final manuscript.
